# Etiology of end-stage liver cirrhosis impacts hepatic natural killer cell heterogenicity

**DOI:** 10.3389/fimmu.2023.1137034

**Published:** 2023-03-30

**Authors:** Ana C. Maretti-Mira, Matthew P. Salomon, Angela M. Hsu, Lily Dara, Lucy Golden-Mason

**Affiliations:** ^1^ USC Research Center for Liver Disease, Keck School of Medicine, University of Southern California, Los Angeles, CA, United States; ^2^ Division of Gastrointestinal and Liver Diseases, Department of Medicine, Keck School of Medicine, University of Southern California, Los Angeles, CA, United States

**Keywords:** natural killer cells, innate lymphocyte cells, NASH, HCV, PSC, cirrhosis, liver, ScRNA-seq

## Abstract

The natural killer (NK) cell population is a critical component of the innate immune compartment of the liver, and its functions are deeply affected by the surrounding environment. In the late stage of fibrosis, NK cells become dysfunctional, but the influence of disease etiology on NK cell behavior during cirrhosis remains unclear. Using single-cell RNA sequencing (scRNA-seq), we characterized the hepatic NK cells from end-stage cirrhotic livers from subjects with non-alcoholic steatohepatitis (NASH), chronic hepatitis C infection (HCV) and primary sclerosing cholangitis (PSC). Here, we show that although NK cells shared similar dysfunctions, the disease etiology impacts hepatic NK cell heterogeneity. Therapeutical strategies targeting NK cells for the prevention or treatment of fibrosis should consider liver disease etiology in their design.

## 1 Introduction

The liver has a crucial homeostatic role in the body’s immunity, harboring a unique immune cell compartment, accurately balancing immune tolerance and activation (1). When not well controlled, chronic liver diseases of different etiologies can progress from fibrosis to cirrhosis and hepatocellular carcinoma (HCC), the leading causes of morbidity and mortality among chronic hepatic patients. Cirrhosis disrupts the hepatic immune stability, compromising the hepatic immune surveillance abilities and eliciting systemic inflammation and immunodeficiency (2). Moreover, cirrhosis disturbs the gut-liver axis, intensifies microbial exposure, and favors the development of a proinflammatory hepatic environment, increasing susceptibility to infection (3).

Natural Killer (NK) cells belong to the innate lymphocyte population in the liver and are particularly abundant since their ratio over total lymphocytes can be up to 5 times higher in the liver than in the peripheral blood (4, 5). These cells are known for their critical participation in early defense against infected and malignant cells through cytotoxicity or cytokine secretion, shaping innate and adaptive immunities (6). Moreover, NK cells are also involved in immune tolerance since they recognize and differentiate “self” versus “non-self” or “altered self” (7). Recently, the NK cells became part of the innate lymphoid cells (ILCs) family, a heterogeneous group of tissue-resident innate lymphocytes ranked into three subsets (8). Group 1 (ILC1s) correspond to NK cells and non-cytotoxic helper ILC1 cells. These two cell types share several features, such as IFN-γ and TNF-α production, cell surface receptors, and immunity against intracellular pathogens. Group 2 (ILC2s) promotes type 2 inflammation and tissue repair, while group 3 (ILC3s) participates in antibacterial immunity, chronic inflammation, and tissue repair. In the liver, the hepatic NK (He-NK) cell population comprises resident NK (rNK) cells, mainly cytokine secretors, and infiltrating conventional circulating NK (cNK) cells, which are predominantly cytotoxic. Accumulating evidence supports an active anti-fibrotic function for He-NK cells (9). NK cells control liver fibrosis by inducing apoptosis of early activated hepatic stellate cells (HSC), the primary drivers of fibrosis, *via* IFNγ-, TRAIL-, NKG2D- and NKp46- dependent mechanisms (10, 11). However, NK cells show dysfunctional behavior during the advanced stages of fibrosis, with a marked reduction of NK cell effector function and increased cell exhaustion, worsening the already existent hepatic fibrosis and increasing the chances of carcinogenic development (12, 13).

Although previous studies have described human NK cells in chronic liver diseases to some extent showing that NK cells display distinct behavior according to the hepatic disease stage and etiology (14), the NK cell attributes during advanced hepatic cirrhosis are still elusive. In general, the majority of evidence suggests that NK cells from cirrhotic livers become similarly dysfunctional in the late stages of fibrosis independent of etiology (6). Here, we propose that hepatic NK cell population heterogeneity is directly correlated to the cause of cirrhosis. We evaluated He-NK cells from end-stage liver diseases of distinct etiologies: non-alcoholic steatohepatitis (NASH), hepatitis C virus infection (HCV), and primary sclerosing cholangitis (PSC). NASH is a sterile inflammatory disease induced by hepatic cellular injury and necroinflammation due to abnormal hepatic lipid accumulation (15). NASH can progress to cirrhosis, liver failure, and hepatocellular carcinoma (16). HCV is a slow and progressive disease caused by a single-stranded hepatotropic RNA virus that commonly starts as an acute infection and progresses to a persistent chronic infection in most individuals. About 10%-20% of the patients advance to cirrhosis (17). PSC is a chronic liver disease characterized by progressive peribiliary inflammation and fibrosis that results in biliary cirrhosis in most cases (18). PSC causes are not well defined. It has been described as an autoimmune disease, a genetic disorder, an inflammatory disease triggered by infectious agents, and a cholangiopathy (19).

Single-cell transcriptomic profiling allowed us to identify the unique and shared features of liver-derived NK cells across these diseases, improving our understanding of NK cell dysfunctionality in advanced liver fibrosis/cirrhosis.

## 2 Materials and methods

### 2.1 Patient selection

In this study, we included liver samples from de-identified patients undergoing liver transplantation due to end-stage liver disease. All subjects had advanced cirrhosis caused by different etiologies: four subjects had NASH, four patients had chronic HCV infection, and four individuals had PSC. We also included control liver samples (no signs of liver inflammation, steatosis, or fibrosis) from four deceased individuals. This study was approved by the University of Southern California Institutional Review Board (HS-18-00254). Written and oral consent was obtained from all enrolled subjects.

### 2.2 Isolation of non-parenchymal cells (NPCs)

Single-cell suspensions of liver NPCs were prepared using mechanical and enzymatic dissociation as previously described (20). Briefly, small pieces from the liver were minced finely using two sterile scalpels, and 20mL of the minced liver was transferred to 50mL tubes containing 30mL of digestion media, (0.05% Collagenase type IV and 0.02% DNase in RPMI 1640). The tube was placed on a rocker and incubated at 37˚C for 2 hours. After digestion, samples were filtered with 100µm strainers and centrifugated at 20xg for 2 mins. The supernatant was transferred to a new tube and centrifuged at 350xg for 10 min at RT. The cell pellet was washed twice with DNAse Wash solution (2% FBS, 1% L-glutamine, 2% antibiotic & antimycotic, 0.02% DNAse, in RPMI 1640) and washed a third time with RPMI medium. Cells were viably frozen at a density of 20-50 million cells/mL.

### 2.3 Natural killer cell isolation by flow cytometry sorting

We thawed frozen NPCs in RPMI with 1% DNAse and centrifuged cell suspensions at 350xg for 5 min at 4°C. Cell suspensions were treated with Debris Removal Solution according to the manufacturer’s instruction (cat. no. 130-109-398, Miltenyi, Auburn, CA). Cells were then incubated at 4˚C in the dark with an antibody cocktail (**Table 1**) for total NK cell selection. After washing, cells were resuspended in PBS+2% FBS and sorted using the BD FACSAria™ Fusion into 90% FBS solution at 4˚C. Sorted cells were centrifuged at 400xg for 5 min at 4°C, resuspended in 50µl of PBS with 0.04% BSA, and cell number and viability were accessed by Countess II (Invitrogen). The gating strategy is shown in **Supplementary Figure 1**.

**Table 1:** Antibodies used for Natural Killer cells FACS sorting.

Antibody	Distributor	Cat. No.
LIVE/DEAD™ Fixable Green Dead Cell Stain kit	Invitrogen	L34970
CD45-APC	eBioscience	17-9459-42
CD3-PE.CF594	BD Horizon™	562280
CD56-V450	BD Horizon™	560360

### 2.4 Single-cell RNAseq

Single-cell suspensions were processed using Chromium Next GEM Single Cell 3’ Kit v3.1 (10x Genomics, Pleasanton, CA). Briefly, up to 16,500 cells were resuspended in the reaction mix and loaded into the Chromium Next GEM Chip G, followed by loading the 3ʹ v3.1 Gel Beads and the partitioning oil. Chip G was placed into the 10x Chromium controller for cell partitioning. After partitioning, 100µL of GEMs (Gel Bead-In EMulsion) were transferred to a new tube and placed at the C1000 Touch Thermal Cycler for the first phase of reverse transcription. All gene expression libraries were prepared and sequenced together to avoid batch effect. Deep sequencing was performed by the USC Molecular Genomics Core, using the Illumina NovaSeq6000 platform. At least 40,000 reads were obtained per cell.

### 2.5 Bioinformatics analyses

FASTQ files were processed using the Cell Ranger count (version 5.0.1, 10X Genomics) pipeline with the GRCh38 (version refdata-gex-GRCh38-2020-A, 10X Genomics) human genome reference to generate gene by count matrices for each sample.

The gene expression count data were then processed using the R package Seurat (version 4) (21). Low-quality cells were removed from the data set by filtering out cells with 1) less than 500 reads, 2) less than 500 or greater than 5,000 genes detected, and 3) cells with greater than 10% of reads mapping to mitochondrial genes.

After filtering out low-quality cells, all samples were integrated into a unified data set using the SCTransform reference-based integration workflow implemented in Seurat (22, 23). The four normal samples were used as the reference data set for the FindIntegrationAnchors function. Differences in mitochondrial gene content were corrected during sample integration by passing the percent of reads mapping to mitochondrial genes to the vars.to.regress parameter in the SCTransform function.

The first 30 PCs were used as the number of dimensions for both the FindNeighbors and FindClusters functions. To find the optimal cluster resolution value we explored a range of resolution values from 0 to 1 by increments of 0.1. We then visualized the range of cluster resolutions by constructing a cluster tree using the R package clustree (24). A final resolution value of 0.2 was used for downstream analyses. Clusters were visualized in two-dimensional space using the RunUMAP function.

Cells were annotated manually by examining the expression of key marker genes among clusters and visualized using the Nebulosa R package (25). Differentially expressed genes between each disease group and normal samples were detected using the FindMarkers function in Seurat with an adjusted p-value of 0.1 as the significance threshold. Expression signatures were calculated using the AddModuleScore function in Seurat using the set of markers genes from Hao et al., 2021 (26).

Ingenuity Pathway Analysis (IPA) software (v01-20-04, Qiagen) was used to determine the canonical pathways and biological processes altered in the different subsets of NK cells (27). The g:GOSt tool from the g:Profiler platform was used to identify the enrichment of biological processes using the differentially expressed genes (28).

RNA velocity: For each sample, spliced and un-spliced RNA counts were generated using functions in the Velcyto (29) package to convert the Cellranger generated bam files to loom formatted files. The loom files were then converted to AnnData objects using the python package Scanpy (30). The UMAP embeddings from the Seurat analysis were incorporated into the AnnData objects. RNA velocities were estimated using functions in the scVelo (31) package and visualized as velocity streams. Top velocity genes were identified using the “rank_velocity_genes” function in scVelo.

### 2.6 Data availability

The data discussed in this publication have been deposited in NCBI’s Gene Expression Omnibus and are accessible through GEO Series accession number GSE217968.

### 2.7 Statistical analyses

All statistical analyses, graphs, and heatmaps were generated using GraphPad Prism version 9 for macOS (GraphPad Software, www.graphpad.com). We used the Kruskal-Wallis test to detect differences between the NK cell subpopulation ratios and the 2-way ANOVA to calculate differences in cell cluster frequencies.

## 3 Results

### 3.1 Single-cell transcriptomic characterization of hepatic natural killer cells in end-stage human liver diseases

**Figure 1:**
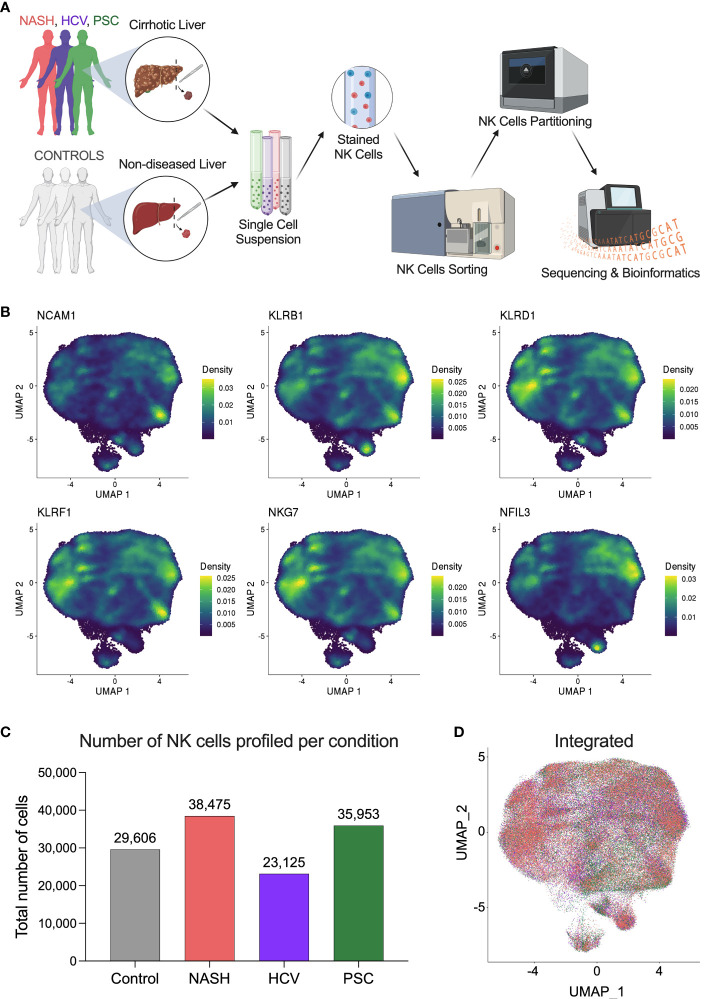
Single-cell RNAseq profiling of human hepatic natural killer cells from end-stage chronic liver diseases. **(A)** Outline of the study experimental design. Liver fragments were processed into single-cell solutions, He-NK cells were FACS-sorted and partitioned for scRNAseq using 10x Genomics technology. Gene expression libraries were sequenced using Illumina platform and processed bioinformatically (Created with BioRender.com). **(B)** UMAPs show the density of cells expressing the classic markers used to confirm the identity of hepatic natural killer cells. **(C)** Total number of NK cells profiled in each group (n=4/condition). **(D)** Integrated UMAP shows the projection of He-NK cells from non-diseased controls (gray), NASH patients (salmon), HCV subjects (purple), and PSC patients (green).

To investigate the heterogeneity of hepatic natural killer (He-NK) cells from advanced cirrhotic livers, we selected patients undergoing liver transplantation due to end-stage liver disease of different etiologies (NASH, HCV, and PSC). As controls, we included hepatic NK cells from non-diseased livers. We included four subjects in each group. The NK cells were sorted by flow cytometry from frozen hepatic non-parenchymal cells (NPCs) and partitioned for scRNAseq using 10x Genomics high-throughput technology (**Figure 1A**, **Supplementary Figure 1**).

NK cell identity was confirmed by gene expression of the classic markers NCAM1, KLRB1, KLRD1, KLRF1, NKG7, and NFIL3 (**Figure 1B**). After quality control and filtering out doublets and other cell types that were not annotated as NKs (ranging from 1 to 3.5% of total called cells), we profiled 29,606 NK cells from controls; 38,475 NK cells from NASH patients; 23,125 NK cells from HCV subjects; and 35,953 NK cells from PSC patients (**Figure 1C**). The integrated Uniform Manifold Approximation and Projection (UMAP) plot shows that the NK cells from the different groups display similar profiles (**Figure 1D**).

**Figure 2:**
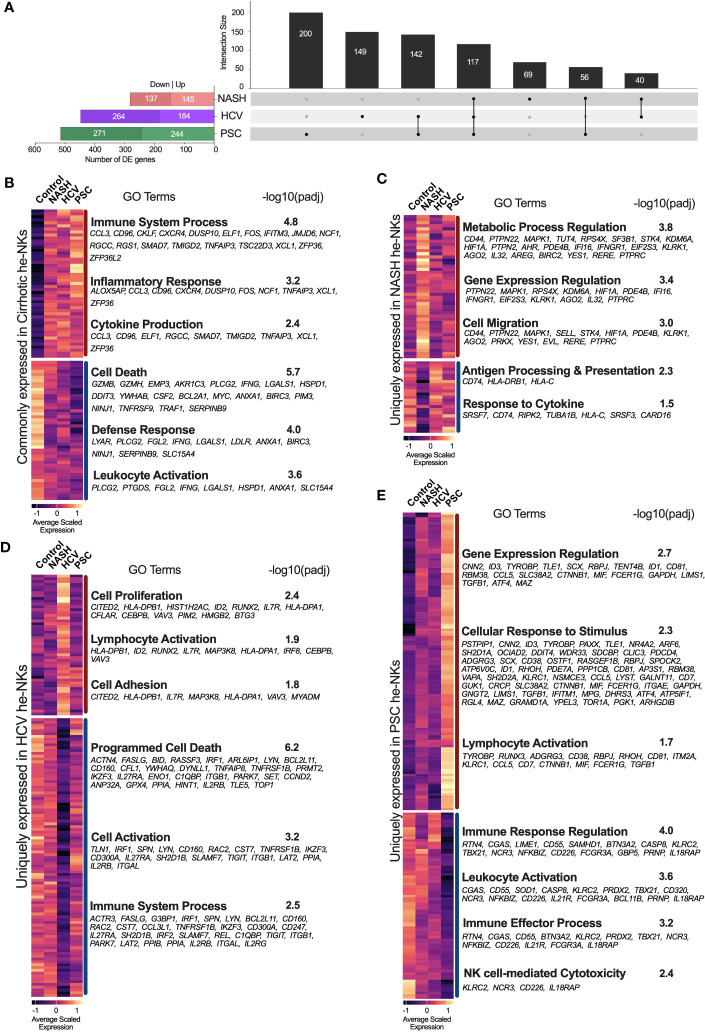
Transcriptional fingerprints of He-NK from end-stage chronic liver diseases. **(A)** Left bars show the total number of differentially expressed genes (DEGs) found in each disease group. Black columns show the intersection of DEGs among He-NKs from cirrhotic livers. We observed that a total of 117 genes were commonly found in cirrhotic livers independent of etiology, while 200 genes were unique for the PSC-derived He-NKs, 149 genes for the HCV He-NKs and 69 genes for the NASH He-NKs. **(B)** Heatmap of the genes commonly expressed by He-NK cells from end-stage cirrhotic livers. GO terms enriched using the commonly 49 upregulated genes (top) and the commonly 46 downregulated genes (bottom). **(C)** Heatmap shows the scaled expression and the GO enriched terms of the uniquely modified genes for NASH, where 46 genes were upregulated, and 23 genes were downregulated. **(D)** Heatmap shows the uniquely modified genes expressed by He-NK cells from HCV patients. GO terms enrichment used 50 upregulated genes (top) and 99 downregulated genes (bottom). **(E)** Heatmap and GO terms of the unique modified genes found in He-NK cells from PSC subjects. We evaluated 123 upregulated genes and 77 downregulated genes. Dark red line: upregulated genes. Dark blue line: downregulated genes.

Calculation of differentially expressed genes (DEG) was based on the comparison between the liver pathology (NASH, HCV, or PSC) and the controls (non-diseased livers). First, we compared the DEGs from total He-NK cells, and we found that the PSC group exhibited the most significant differences in NK global gene expression, with a total of 515 DEGs (244 up and 271 downregulated), followed by the HCV group, with 448 DEGs (184 up and 264 downregulated) and then the NASH group, with 282 DEGs (145 up and 137 downregulated) (**Figure 2A**). The intersection of DEGs among the diseases identified 117 genes commonly expressed by all disease groups (**Figure 2B**). From them, 49 genes were upregulated and enriched biological processes related to immune and inflammatory responses, while 46 genes were downregulated and enriched events like cell death, cell activation, and defense response. Notable upregulated genes with immunological functions included CXCR4, CCL3, and GZMK. Among the main downregulated genes of immunological importance, we found GZMB, GZMH, and IFNG.

We also determined the transcriptional fingerprints (uniquely modified transcripts) of He-NK cells from end-stage liver diseases. In NASH, we found 69 unique DEG, most of which were upregulated (46 genes) and correlated with the metabolic process of gene expression regulation, and cell migration. The other 23 genes (downregulated) were related to antigen processing and presentation and response to cytokines (**Figure 2C**). In the HCV group, we detected 149 unique DEG, with 50 genes upregulated linked to cell proliferation and adhesion, as well as lymphocyte activation; and 99 genes downregulated related to cell activation, cell death, and immune response (**Figure 2D**). The PSC group displayed 200 unique DEGs, with 123 upregulated genes involved in gene expression, cellular response to stimulus, and lymphocyte activation; and 77 downregulated genes involved in immune response, leukocyte activation, and cytotoxicity (**Figure 2E**).

### 3.2 Natural killer cell subpopulation frequencies vary according to disease etiology

**Figure 3:**
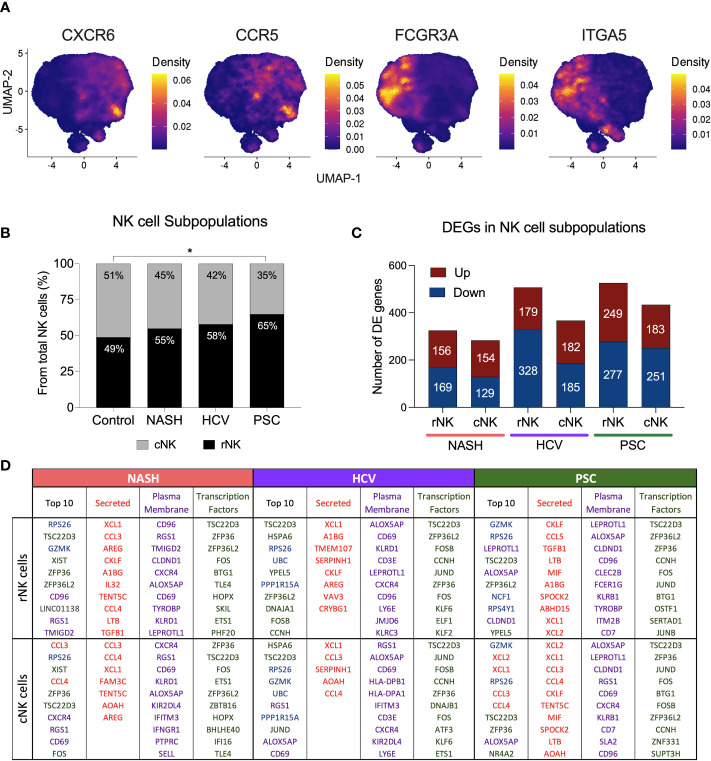
Hepatic Resident and Infiltrating NK cells profiling in end-stage chronic liver diseases. **(A)** UMAPs showing the density of cells expressing CXCR6, ITGA5 (CD49e), CCR5 and FCGR3A (encoding CD16). NK cells CXCR6^+^ CCR5^+^ ITGA5^-^ and FCGR3A^-^ are identified as resident NK cells (rNK), and NK cells FCGR3A^+^ ITGA5^+^ CXCR6^-^ CCR5^-^ are classified as conventional infiltrating NK cells (cNK). **(B)** Analysis of cNK/rNK cells frequencies in the 4 groups indicated an increase of rNK subpopulation in cirrhotic livers. The PSC group has significantly more rNK cells than the healthy group (* p<0.05). **(C)** Numbers of differentially expressed (DE) genes found in each subpopulation among diseases. **(D)** Top ten most highly expressed DE genes in hepatic rNK and cNK cells from NASH, HCV, and PSC subjects. Upregulated genes were ranked by P-value among the total gene set and among genes encoding secreted proteins, plasma membrane proteins and transcription factors. Secreted proteins were color-coded in red, plasma membrane protein-encoding genes are purple, transcription factor-encoding genes are green, genes encoding cytoplasmic protein are in blue and genes encoding other proteins are gray.

We defined hepatic NK cell subpopulations based on the expression of CXCR6, ITGA5, CCR5, and FCGR3A (**Figure 3A**, **Supplementary Figure 2**). The two major He-NK cells groups are known as liver resident NKs (rNKs), described as CXCR6^+^ CCR5^+^ ITGA5^-^ FCGR3A^-^; and infiltrating conventional circulating NK cells (cNKs), characterized as FCGR3A^+^ ITGA5^+^ CXCR6^-^ CCR5^-^. We assessed the frequencies of each subpopulation across the disease states and found that the ratio of He-NK cell subpopulations in control subjects was well-balanced, with 49% rNK cells and 51% cNK cells, corroborating the findings of other authors (14). In cirrhotic livers, however, we observed a predominance of the rNK cell subpopulation over the cNK cells, especially in the PSC group, where 65% of total NK cells were rNK cells. The HCV and NASH groups also showed a change in the cNK/rNK ratios, with 58% and 55% of rNK cells among total NK cells, respectively (**Figure 3B**).

We calculated the number of DEG in each NK cell subpopulation from NASH, HCV, and PSC cirrhotic livers (**Figure 3C**). In NASH, we found 325 DE genes in rNK cells (156 up, 169 downregulated), and 283 DE genes in cNK cells (154 up, 129 downregulated). In HCV, we detected 507 DE genes (179 up, 328 downregulated) in rNK cells and 367 DE genes (182 up, 185 downregulated) in cNK cells. In PSC, we identified 526 DE genes (249 up, 277 downregulated) in rNK cells and 434 DE genes (183 up, 251 downregulated) in cNK cells. We identified the top ten upregulated genes in each group (**Figure 3D**). In rNK cells from NASH, we found GZMK and the inhibitory receptor CD96 were upregulated, while cNK cells overexpressed the chemokines CCL3 and CCL4, the activation marker CD69, and the chemokine receptor CXCR4. In HCV, the top genes overexpressed by rNK cells encode several proteins involved in cellular responses to stress (HSPA6, RPS26, UBC, PPP1R15A, and DNAJA1), while the cNK cells, besides the stress response genes, also had higher expression of GZMK and ALOX5AP (Arachidonate 5-Lipoxygenase Activating Protein). In PSC, the top upregulated genes in rNK cells were GZMK, ALOX5AP, and NCF1 (Neutrophil Cytosolic Factor 1), and in cNK cells we found genes encoding proteins associated with NK cell effector functions (GZMK, XCL2, XCL1, CCL3, and CCL4), and ALOX5AP to be elevated. The top ten most expressed genes for secreted proteins, plasma membrane proteins, and transcription factors can also be found in **Figure 3D**.

### 3.3 Infiltrating conventional He-NK cells from NASH and PSC end-stage liver diseases are better cytokine expressors

**Figure 4:**
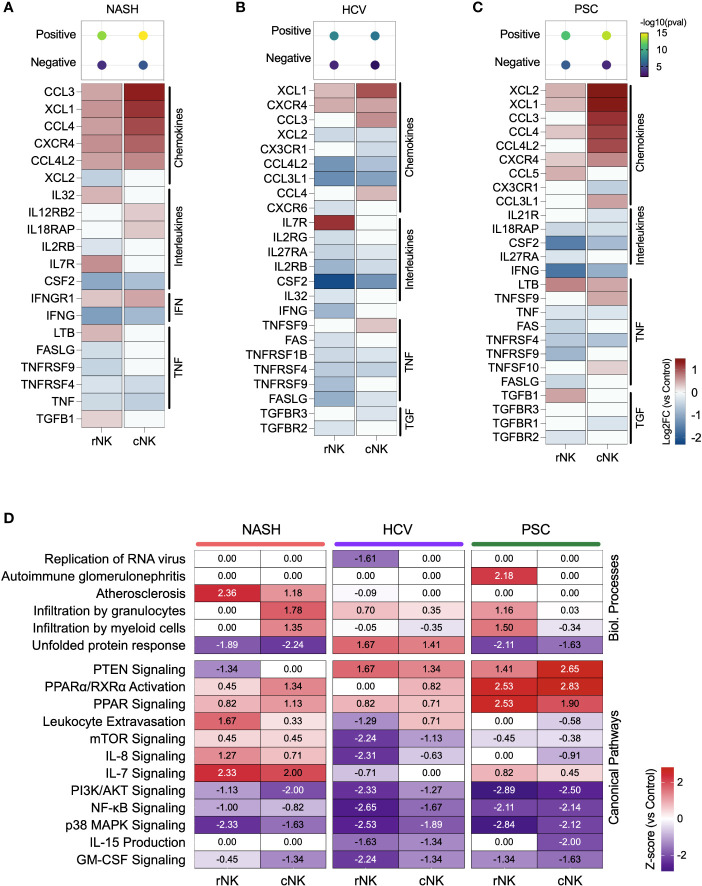
Inflammatory activity of hepatic NK cells in end-stage liver diseases. **(A–C)** Top: enrichment scores for positive and negative regulation of cytokine production. Enrichment scores are scaled by -log10(pval). Bottom: fold changes (log2) of cytokines and their receptors expressed by NASH **(A)**, HCV **(B)**, and PSC **(C)** He-NKs compared to healthy He-NK cells. All values are expressed in the same scale and are statistically significant (Enrichment scores: -log10(pval) > 1.3; DEG: Log2FC > 0.27, FDR < 0.1). **(D)** Heatmap showing the most significant biological processes and canonical pathways related to inflammation and cytokines in He-NKs. Z-score values are included inside the heatmap.

We calculated the positive or negative regulation of cytokine expression using the g:GOSt (g: Profiler) enrichment test with the DEG lists. Our dataset revealed a stronger positive regulation of cytokine expression in the cNK subpopulations from NASH and PSC cirrhotic livers. He-NK cells from HCV patients demonstrated the lowest positive cytokine production scores (**Figures 4A–C**, top).

Regarding the cytokine/receptor repertories, cNK cells from NASH livers expressed the receptors IL12RB2, IL18RAP, and IFNGR1. The rNK cells overexpressed IL-32, LTB, and TGFB1. Both subpopulations expressed CCL3, CCL4, CCL4L2, and XCL1, but cNK cells expressed them at higher levels (**Figure 4A**). In HCV, cNK cells overexpressed CCL3, CCL4, and TNFSF9. Both subpopulations expressed XCL1 and the receptor CXCR4 (**Figure 4B**). In PSC, rNK cells expressed high levels of CCL5 and TGFB1, while cNK cells overexpressed CCL3, CCL4L2, CCL3L1, TNFSF9, and TNFSF10. Both populations expressed XCL1, XCL2, CCL4, and LTB, with cNK cells expressing the highest levels (**Figure 4C**).

The z-scores of biological processes and pathways related to inflammation in the hepatic NK cells are shown in **Figure 4D**. The main difference observed in He-NKs from NASH livers is that cNK cells showed higher z-scores for granulocyte infiltration, while rNK cells upregulated leukocyte extravasation and had a stronger involvement in atherosclerosis. Both subpopulations were upregulated for IL-7 signaling. He-NKs from HCV livers showed upregulation of the unfolded protein response and PTEN signaling pathways. HCV-derived rNK cells showed inhibition of RNA virus replication and inflammatory pathways such as mTOR, PI3K/AKT, and NF-κB signaling. PSC-derived rNK and cNK cells demonstrated upregulation of the anti-inflammatory pathways PTEN and PPAR signaling and downregulation of pro-inflammatory events such as PI3K/AKT, and NF-κB signaling suggesting that both subpopulations display anti-inflammatory profiles. Additionally, rNK cells from PSC livers displayed a strong upregulation of the autoimmune glomerulonephritis biological process.

### 3.4 Cytotoxicity scores are increased in cNK cells from NASH cirrhotic livers

**Figure 5:**
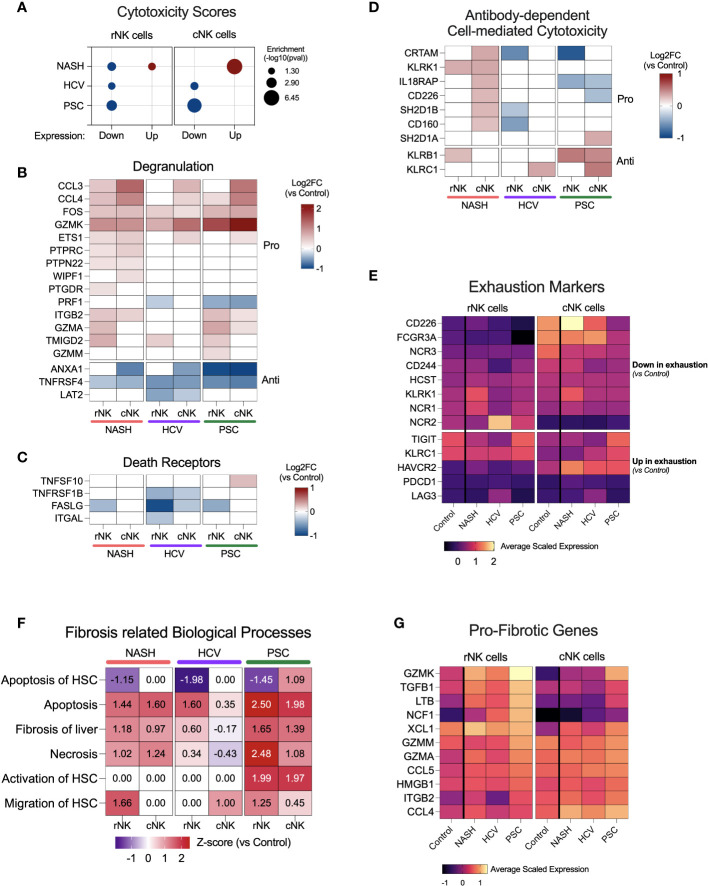
NK cell cytotoxicity and contribution to fibrosis in end-stage cirrhotic livers. **(A)** Enrichment scores for cytotoxicity using up or downregulated genes from rNK and cNK cell datasets. In NASH-derived cNK cells, we observed that the upregulated genes significantly enrich cytotoxic pathways. In HCV and PSC, only the downregulated genes were significantly enriched for cytotoxic events, suggesting downregulation of NK cell cytotoxicity in these two diseases. **(B)** Heatmap showing fold changes (log2) of genes related to degranulation in He-NK cells. **(C)** Fold changes (log2) of most relevant genes involved in cytotoxicity *via* death receptors. **(D)** Fold change (log2) of genes related to antibody-dependent cytotoxicity. **(E)** Average scaled expression of genes related to NK cell exhaustion reveals that cNK cells from PSC patients showed the strongest pattern of exhaustion compared to cNK cells from healthy livers. **(F)** Main biological processes and canonical pathways related to fibrosis. Z-score values are included inside the heatmap. **(G)** Average scaled expression of the most important pro-fibrotic genes expressed by rNK and cNK cells.

We used the up or downregulated DE gene lists to analyze cytotoxicity pathways. We found that the upregulated DE genes from NASH-derived cNK cells showed the most robust enrichment of cytotoxicity (**Figure 5A**). rNK cells from NASH showed significant enrichment of cytotoxicity among upregulated and downregulated genes, which suggests that cytotoxic activity was not significant in these cells. The genes significantly enriching cNK and rNK cells’ cytotoxicity in HCV and PSC subjects were downregulated.

Cytotoxicity in NK cells may take place *via* three pathways: degranulation, death receptors, and antibody-dependent cell-mediated cytotoxicity (ADCC). The genes involved in the degranulation pathway were upregulated mainly in He-NK cells from the NASH group (**Figure 5B**). He-NK cells from PSC patients showed upregulation of gene coding for the granzymes GZMK, GZMA, and GZMM. Although these cells showed a robust upregulation of other genes involved in degranulation, the downregulation of PRF1 in both subpopulations indicates a less efficient cytotoxicity mediated by granules. Nevertheless, cNK cells from PSC are the only cell group that overexpressed TNFSF10 (encoding TRAIL), a key mediator for cytotoxicity induced by the death receptors (**Figure 5C**), suggesting the importance of this pathway for PSC-derived cNK cell cytotoxicity. ADCC was upregulated in cNK cells from the NASH group (z-score = 2.1), and the main genes related to this process are shown in **Figure 5D**. Overall, our findings suggest a distinct pattern of cytotoxicity in NK cell subsets from cirrhotic human livers according to the hepatic disease etiology.

Considering the reduced expression of IFNG, TNF, and GZMB and the low scores for cytotoxicity, we decided to evaluate the expression of markers for NK cell exhaustion (**Figure 5E**), which is characterized by the downregulation of activating receptors and upregulation of inhibitory receptors, besides upregulation of PD-1 (PDCD1) and LAG3, in comparison to NK cells from control livers. We found that cNK cells from PSC showed the strongest pattern for exhaustion, with downregulation of CD226, FCGR3A, and NCR3, and upregulation of TIGIT, KLRC1, and HAVCR2 (encoding Tim-3) when compared to control cNK cells.

### 3.5 Liver resident NK cells are involved in liver fibrosis

Several lines of evidence suggest that NK cells are important to decide fibrosis fate. The biological processes related to fibrosis enriched by the DEG lists showed upregulation of fibrosis of the liver, necrosis, and NK apoptosis in most of the NK subpopulations, especially in the PSC subjects (**Figure 5F**). Remarkably, apoptosis of hepatic stellate cells (HSC) was downregulated in the rNK cells from NASH, HCV, and PSC livers, mainly due to the significant downregulation of FASLG and IFNG (**Figures 4A–C**). In PSC, we also found that both rNK and cNK cells were upregulated for activation of HSC. Migration of HSCs was also upregulated in rNK cells from the NASH group.

**Figure 5G** shows a heatmap with several genes significantly expressed in our dataset with pro-fibrotic function. They are GZMK, TGFB1, LTB, NCF1, XCL1, GZMM, GZMA, CCL5, HMGB1, ITGAB2, and CCL4. We found that these genes were mostly upregulated in cirrhotic rNK cells when compared to non-diseased controls.

### 3.6 Single-cell RNAseq maps eight distinct hepatic NK cell subsets in human livers

**Figure 6:**
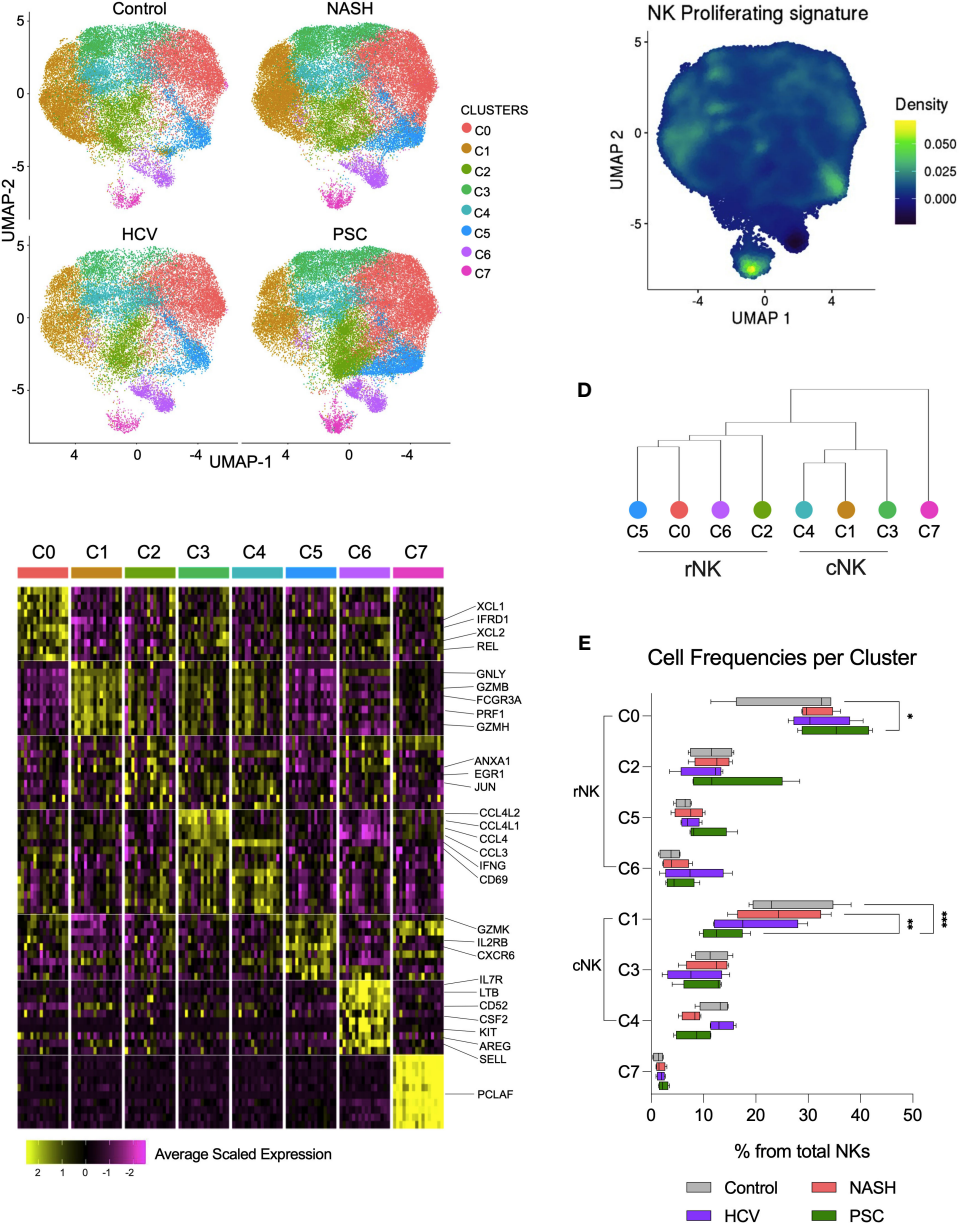
Single-cell RNAseq revealed eight subsets of hepatic NK cell in end-stage liver diseases. **(A)** UMPAs showing He-NK cells clusters in healthy, NASH, HCV, and PSC livers. **(B)** The main transcriptomic markers observed in each cluster indicated that clusters C0, C2, C5 and C6 are rNK cells, clusters C1, C3 and C4 are cNK cells and C7 displays features of cycling NK cells. **(C)** Dendrogram showing the relationships of NK cell clusters. **(D)** The analysis of cell frequencies per cluster showed significant variation of cells in clusters C0 and C1. *P-value < 0.05; **P-value < 0.01; ***P-value < 0.001.

We deepened the clustering analysis of the He-NK cells and found a total of eight He-NK cell subsets present both in physiological and pathological conditions (**Figure 6A**). Among the most relevant gene overexpressed by each cluster, we highlight XCL1, XCL2, IFRD1, and REL in cluster C0; GNLY, GZMB, FCGR3, PRF1, and GZMH in cluster C1; ANXA1, EGR1, and JUN in cluster C2; CCL4L1, CCL4L2, CCL4, CCL3, IFNG, and CD69 in clusters C3 and C4; GZMK, IL2RB and CXCR6 in cluster C5; IL7R, LTB, CD52, CSF2, KIT, AREG, and SELL in C6; and PCLAF in cluster C7 (**Figure 6B**).

Clusters C0, C2, C5, and C6 overlapped with the rNK cell subpopulation, while clusters C1, C3, and C4 overlapped with the cNK cells (**Figure 3A**, **Supplementary Figure 2**). Cluster C7 overexpressed genes specific for cycling cells, such as MKI67, KLRF1, TYMS, TRDC, TOP2A, FCER1G, PCLAF, CD247, CLSPN, and ASPM (**Figure 6C**), and therefore, it was classified as proliferating NK cells. The hierarchical clustering of the gene expression profiles supported this classification (**Figure 6D**).

We estimated the frequencies of each subset among the total hepatic NK cells and found that the clusters C0 and C1 frequencies vary according to the disease etiology. Cluster C0 significantly increased in PSC, while cluster C1 significantly decreased in PSC livers (**Figure 6E**), supporting the increased predominance of rNK cell subpopulation over cNK cells in PSC, shown in **Figure 3B**. We performed RNA velocity analysis to understand better the cellular transitions underpinning this fluctuation. The hepatic NK cell differentiation flow in non-diseased control livers progresses from the cNK subset C1 to the rNK cluster C0. Clusters C3 and C4 are transient cNK cells deriving from the C1 subset. The RNA velocities of the rNK clusters C2 and C5 also flow toward the C0 subpopulation. However, clusters C6 and C7 do not contribute to other clusters’ formation (**Supplementary Figure 3A**). Evaluating the RNA trajectories in the diseased groups, we observed that in advanced NASH, the flow of cell differentiation goes from clusters C1 and C3 toward cluster C0, both *via* the transient cluster C4. Clusters C2 and C5 also contributed to C0 formation. Differently from the control, cluster C1 shows less intense RNA velocity, with multiple vectors emerging from cluster C7 (**Supplementary Figure 3B**). In advanced cirrhosis caused by HCV infection, we observe RNA velocities directed from the cNK cluster C1 toward the rNK cluster C0 with cluster C4 as a transient state, whereas RNA velocities from cluster C3 contribute directly to cluster C0. We also observed that, like in NASH, the RNA velocity in C1 is slower(**Supplementary Figure 3C**). In end-stage PSC, we detected more intense RNA velocities from C1 to C0, with clusters C3 and C4 as transient subsets. The rNK C0 showed a slower RNA velocity than the control and the other disease groups (**Supplementary Figure 3D**).

**Figure 7:**
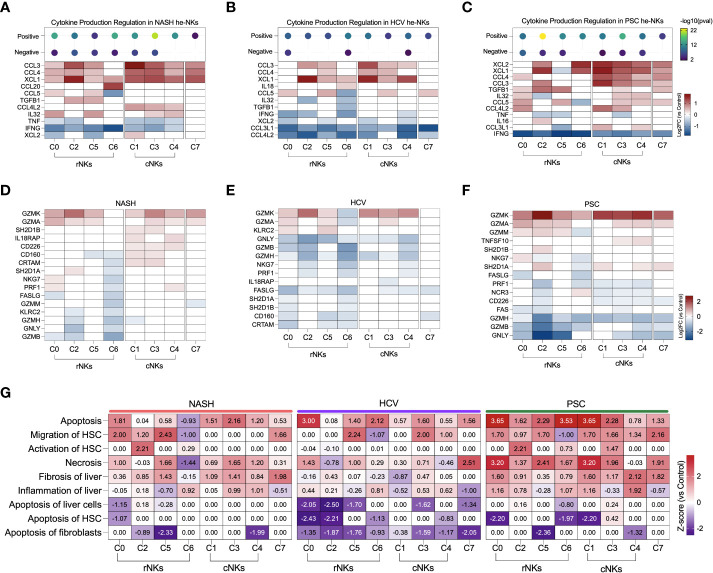
Transcriptomic analysis of NK cell clusters effector functions in end-stage liver diseases. **(A–C)** Top: Enrichment scores for cytokine production regulation. Bottom, main cytokines expressed by NK cells. NASH **(A)**, HCV **(B)**, and PSC **(C)**. **(D–F)** Main genes related to cytotoxicity expressed by NK cells in. NASH **(D)**, HCV **(E)**, and PSC **(F)**. **(G)** Heatmap displaying the main biological processes related to inflammation and fibrosis.

We analyzed the effector functions of the NK cell subsets using the transcriptomic dataset, focusing on the expression of secreted cytokines, cytotoxicity markers, and overall contribution to fibrosis. In NASH, cluster C3 showed the strongest positive regulation of cytokines production (**Figure 7A**). Most of the clusters express CCL3, CCL4, and XCL1 and were inhibited for IFNG and TNF expression. Among the rNK cells, cluster C0 expressed mediators known to participate in inflammation during obesity (CCL5 and IL32). Cluster C2 expressed TGFB1, a pro-fibrotic factor. Cluster C5 expressed genes TGFB1, CCL5, and IL32, suggesting participation in NASH progression. Cluster C6 expressed CCL20, chemokine elevated during obesity. Among the cNK cells, all clusters expressed CCL4L2. The subset C1 also expressed XCL2, and the clusters C3 and C4 expressed IL32.

In He-NK cells from HCV cirrhotic livers, most of the clusters showed a weak positive regulation of cytokine production with inhibition of CCL3L1 and CCL4L2 gene expression (**Figure 7B**). Most of the rNK cell subsets were also inhibited for IFNG while expressing high levels of XCL1. Additionally, cluster C6 expressed IL18, a pro-inflammatory cytokine elevated in HCV cirrhotic patients (32), while cluster C5 expressed CCL3, CCL4, and CCL5. All cNK clusters expressed CCL3 and XCL1. Cluster C1 expressed CCL4, and cluster C3 expressed CCL4 and CCL5, showing the importance of these subsets in the recruitment of cells. Cluster C7 expressed CCL5.

The clusters C2 and C3 in PSC displayed the highest scores for positive regulation of cytokine production (**Figure 7C**). The majority of the rNK subsets expressed TGFB1, except C6. Cluster C0 expressed cytokines involved in liver inflammation (CCL4, IL32, CCL5, CCL4L2), and cluster C2 expressed the chemoattractants XCL1 and XCL2, CCL3, and IL16. Cluster C5 expressed CCL5, and cluster C6 expressed XCL1, and XCL2. All cNK clusters expressed XCL1, XCL2 CCL4, and CCL3. Cluster C3 was the cluster with the highest number of cytokines expressed, including TGFB1, IL32, CCL5, and CCL4L2. Cluster C4 expressed IL32, CCL5, and CCL3L1. The cycling NK expressed more cytokines in PSC than in NASH or HCV.

Regarding cytotoxicity, the granzymes A and K were the most expressed by the NK cell subsets among all diseases (**Figures 7D–F**). In NASH, clusters C0 and C4 expressed more PRF1 than the control group (**Figure 7D**). Clusters from the cNK cell subpopulation overexpressed genes that favor ADCC (IL18RAP, CD160, CRTAM, SH2D1A). Among the etiologies, He-NK cell subsets from HCV subjects showed the strongest downregulation of cytotoxicity markers (**Figure 7E**). In PSC, cNK cell clusters C3 and C4 expressed TNFSF10, encoding TRAIL (**Figure 7F**).

The biological processes associating He-NK cell subsets with liver fibrosis can be found in **Figure 7G**. Necrosis, liver fibrosis, and inflammation were positively regulated in several clusters, with the highest z-scores in PSC NK cell subsets.

Cell apoptosis was strongly upregulated in all the diseases, but not always in the same subsets of NK cells. Interestingly, activation of HSC was significantly upregulated in cluster C2 in NASH and PSC groups, while induction of HSC apoptosis was strongly inhibited in HCV (C0 and C2) and PSC (clusters C0, C6, and C1) subjects.

These findings support the different functions that each NK cell subset has in the cirrhotic livers and that those functions are also different according to the disease etiology.

### 3.7 Identification of innate lymphoid cells in cirrhotic livers

**Figure 8:**
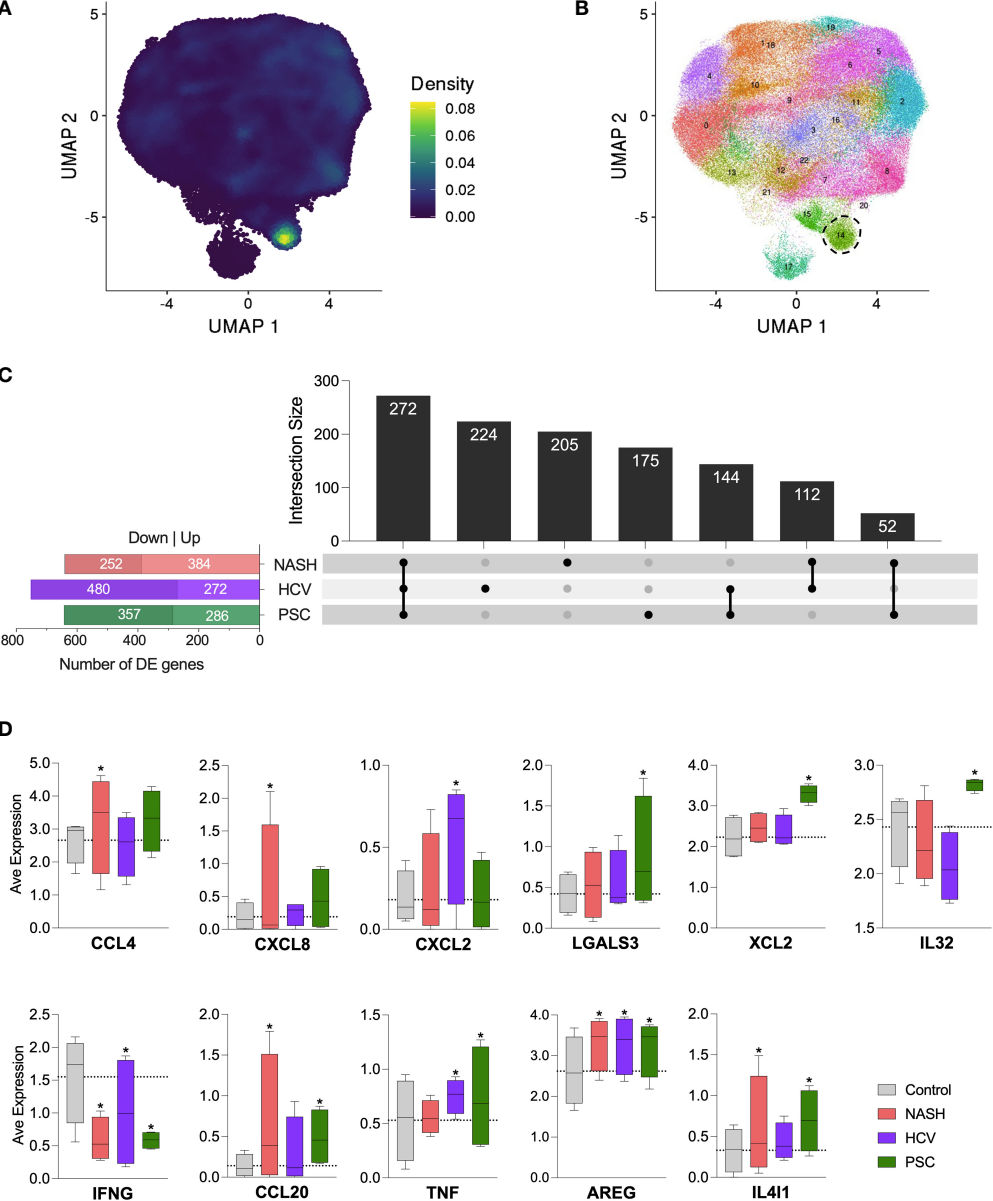
Detection of innate lymphoid-like cells (ILC-like) among hepatic NK cells in cirrhotic livers. **(A)** UMPA showing the ILC transcriptomic signature density among the cell clusters. **(B)** Increase of clustering analysis resolution showed that subset C6 subdivided into two clusters. Cluster surrounded by dot lines indicate ILC-like cells. **(C)** Differentially expressed genes identified in the ILC-like cluster and their intersections. **(D)** Average normalized expression of DE genes with immunological significance. * Significant difference compared to control cells according to DEG calculation.

Since cluster C6 displayed a high expression of the genes encoding CD127 (IL7R) and c-Kit (KIT), important markers of innate lymphoid cells (ILCs) (33), we evaluated our dataset with an ILC signature based on the expression of KIT, TRDC, TTLL10, LINC01229, SOX4, KLRB1, TNFRSF18, TNFRSF4, IL1R1, HPGDS (**Figure 8A**). We identified a strong concentration of ILC-like cells as part of cluster C6. When we increased the resolution of our clustering analysis, C6 was subdivided into two clusters, one corresponding to ILC-like cells detected in our dataset (**Figure 8B**). No significant difference in the frequency of ILC-like cells was observed among the diseases (data not shown). We further analyzed the differentially expressed genes within this ILC-like cluster and observed a high number of DE genes in all the diseases (**Figure 8C**). We identified 641 genes in NASH (389 up, 252 downregulated), 752 genes in HCV (272 up, 480 downregulated), and 643 genes in the PSC group (286 up, 357 downregulated). The three groups shared the expression of 272 genes, in general, enriching biological events related to immune response, cell death, and cell activation. The other genes uniquely expressed by ILC-like cells in NASH, HCV, and PSC did not enrich any pathway different than those highlighted by the genes commonly expressed. The DE genes of immunological interest identified in the ILC-like cluster are shown in **Figure 8D**. CCL4 and CXCL8 were upregulated in NASH, CXCL2 was upregulated in HCV, and LGALS3, XCL2, and IL32 were overexpressed in PSC. All groups showed inhibition of IFNG expression and upregulation of AREG. ILC-like cells from NASH and PSC groups were upregulated for CCL20 and IL4I1, and surprisingly TNF was upregulated in ILC-like cells from HCV and PSC subjects.

## 4 Discussion

This study aimed to assess the heterogeneity of hepatic natural killer (He-NK) cells in different end-stage chronic liver diseases. NK cells are an essential component of the hepatic innate immune system, critical for removing pathogens and cancer cells. In addition, the effector function of NK cells can directly promote or constrain liver disease progression. NK cell cytotoxicity toward hepatocytes, cholangiocytes, and other liver cells is employed to achieve tissue homeostasis after an acute liver injury. Conversely, the chronic tissue insult dysregulates the inflammatory response and favors fibrosis development (34). Fibrosis progression is marked by the activation of hepatic stellate cells (HSC) and hepatic macrophages, which secrete high levels of extracellular matrix compounds, unbalancing the restoration of the damaged tissue (35, 36). At this stage, NK cells play a critical role in inducing early activated HSCs to apoptosis *via* cytotoxicity or IFN-γ secretion, thus delaying the progression of fibrogenesis (37). NK cells also contribute to liver fibrosis control through the secretion of several factors that can recruit other immune cells and shape the resulting adaptive immune response (6). As liver fibrosis progresses to cirrhosis, NK cell effector functions may become compromised: NK cells decrease the expression of cytokines, activating receptors, and degranulation capacity, allowing the permanence of activated HSCs and cirrhosis progression (38). It is unknown if dysfunctional hepatic NK cell responses in end-stage liver disease correlate to specific pathologies or merely reflect the cirrhotic microenvironment. Several factors in the liver microenvironment could affect NK cell functionality, such as inflammation level, fibrosis score, NK cell distribution, and overall liver dysfunction. Currently, we lack studies that examine the effector profiles of intrahepatic NK cells from livers in the same stage of dysfunction due to different etiologies. Therefore, our study used three distinct chronic hepatic diseases – sterile inflammation (NASH), virus-induced (HCV), and autoimmune-like (PSC) – to investigate whether and how different diseases could influence hepatic NK cells in end-stage cirrhotic liver. All patients included in this study required liver transplantation.

Firstly, we detected an expansion of the resident NK cells subpopulation in livers with end-stage cirrhosis. In non-diseased livers differently from peripheral blood, we find resident and circulating NK cells at similar rates (14). Among the analyzed diseases, PSC showed the highest increase in rNK cell frequency, with 65%, followed by HCV (58%) and NASH (55%). The scRNAseq technique provides a snapshot in time of the transcriptional state of the analyzed cells. We can determine the transcriptional changes within individual cells and predict their developmental trajectories by evaluating the ratios of spliced to unspliced mRNA using RNA velocity analysis. Our results revealed that the circulating NK cells contribute to the formation of the major resident NK cells cluster. Interestingly, this is not an exclusive feature shared by NK cells from the diseased groups. We detected similar trajectories in the non-diseased control group, which suggests that part of the cNK cells recruited to the liver are potentially intrahepatic rNK cell precursors, as already proposed by other authors (39).

The hepatic NK cells profiled in this study displayed several similarities across disease states. The expression of genes needed for NK cell effector functions, such as interferon-gamma (IFN-γ), tumor necrosis factor-α (TNF-α), granulocyte-macrophage colony-stimulating factor (CSF2), granzyme B (GZMB), and FAS ligand (FASLG), was inhibited in the majority of the NK cells from cirrhotic livers. Concurrently, we detected significant overexpression of CCL3, CCL4, and XCL1, which contribute to the recruitment of immune cells, such as monocytes, neutrophils, NK and T cells (40, 41), and XCR1-expressing dendritic cells capable of antigen cross-presentation, an event that may prolong the inflammatory response and favor fibrosis (42). CCL3 and CCL4 neutralization or the deficiency of their receptors, CCR1 and CCR5, provenly attenuate hepatic fibrogenesis and monocyte infiltration in mouse models (43). Therefore, a common feature of NK cells from cirrhotic livers despite the disease etiology is the recruitment of other inflammatory cells that may contribute to fibrosis progression.

**Figure 9:**
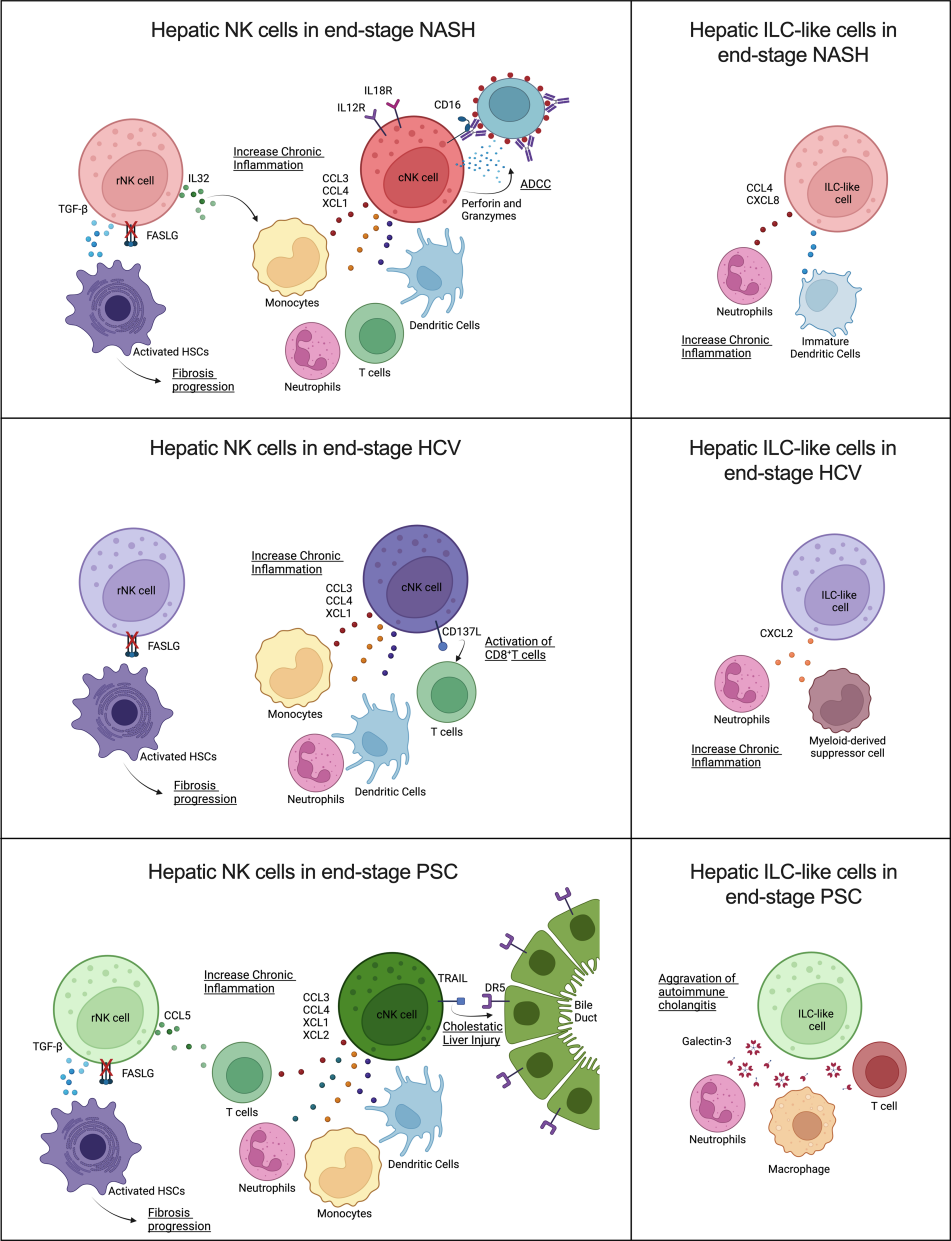
Contribution of human hepatic resident and circulating NK cells and ILC-like cells to end-stage liver diseases caused by **(A)** NASH, **(B)** HCV, and **(C)** PSC. Overall, cNK cells and ILC-like cells may contribute in different ways to chronic inflammation, while rNK cells may favor hepatic fibrosis progression by failure to kill activated hepatic stellate cells (HSC) due to inhibition of FASLG (Created with BioRender.com).

Remarkably, our findings suggested that NK cells derived from livers affected by different diseases also displayed unique features (**Figure 9**).

The participation of NK cells in the progression of NASH remains controversial, and the disease stage can significantly influence the NK cells’ behavior (44). Here we show that, in advanced NASH, cNK cells behave as pro-inflammatory cells. These cells expressed high levels of chemoattractants (CCL3, CCL4, and XCL1) of other immune cells that can exacerbate NASH-associated inflammation, and overexpressed IL12 and IL18 receptors, a fact that suggests they are more prone to be activated and participate in the recruitment of CD8^+^ T cells (45). In addition, these cells displayed a robust cytotoxic profile, mainly involving antibody-dependent cell-mediated cytotoxicity (ADCC). NK cell effector functions can evoke ADCC *via* binding CD16 (FCGR3) or CD32c (FCGR2) to the Fc portion of immunoglobulins in opsonized target cells. After binding, NK cells can induce cell death through degranulation, cell death receptors, or the release of pro-inflammatory cytokines (46). Although ADCC is an essential mechanism employed in tumor surveillance and viral clearance, it has also been associated with the progression or worsening of autoimmune and infectious diseases, such as neuromyelitis optica, dengue fever, and influenza, due to immunoglobulin polymorphisms or epitope-specificity antibodies produced by the organism (47–49). However, the beneficial or detrimental participation of ADCC in NASH progression has yet to be studied. The rNK cells in NASH cirrhosis expressed higher levels of TGFB1 and demonstrated inhibition of the HSC apoptosis pathway. As described before, HSC killing is one of the mechanisms used by NK cells to control fibrosis. Besides the TGF-β pro-fibrotic effects on HSCs activation and M2 macrophage polarization, this growth factor also suppresses NK cell effector functions (50). Resident NK cells also overexpressed IL32, a pro-inflammatory cytokine involved in fibrosis progression in NAFLD (51, 52). These findings suggest that rNK cells have a pro-fibrotic role in severe NASH. Moreover, the presence of ILC-like cells expressing CCL4 and CXCL8 may contribute to the effects of the cNK cells in the perpetuation of liver inflammation since these chemokines are chemoattractants for neutrophils and immature dendritic cells (45, 53).

NK cells are critically important in the early immune response for the clearance of viral infections, mainly through the production of IFN-γ and TNF-α. Although HCV infection reportedly inhibits this NK cell effector function, shifting from IFN-γ production toward cytotoxicity in the chronic phase (54), the hepatic dysfunction resulting from the HCV infection directly influences NK cell activation capacity. NK cells isolated from the blood of chronic HCV patients with high ATL levels showed increased *in vitro* degranulation capacity. Additionally, hepatic NK cells from chronic HCV patients that presented altered levels of ALT, mild hepatic inflammation, and low liver fibrosis scores displayed strong cytotoxicity markers, such as NKp46, CD122, and TRAIL (55). However, hepatic NK cells isolated from HCV-infected livers showing altered ATL levels, low scores of necroinflammation, and mild fibrosis, showed impaired degranulation but normal cytokine secretion (56). Our results demonstrated that NK cells isolated from livers with advanced fibrosis resulting from HCV infection lose both their cytokine expression and cytotoxic capacities. Most of the cytokines detected in our dataset were inhibited, mainly in the rNK cell subpopulation, which showed significant inhibition of IFNG. TNF was not significantly different from the controls in both NK cell subpopulations. On the other hand, HCV-associated cNK cells showed a pro-inflammatory profile, with high expression of the chemoattractants CCL3, CCL4, and XCL1, and the ligand TNFSF9 (CD137L), a costimulatory signal that can induce CD8^+^ T cells response against viral infection (57). The ILC-like subset also showed pro-inflammatory features overexpressing CXCL2, a chemokine that participates in the recruitment of neutrophils and myeloid-derived suppressor cells (58, 59), cells that can aggravate chronic inflammation. Of note, the rNK cell subpopulation displayed significant downregulation of several pro-inflammatory pathways, such as mTOR and NF-kB signaling, and of HSC apoptosis, which may implicate this subpopulation in the pro-fibrotic events happening in the liver in end-stage chronic HCV infection.

NK cells play diverse roles in autoimmune disorders (60). Previous studies correlate PSC pathology to the imbalance between inhibitory and activating killer-immunoglobulin receptors (KIRs) ligands observed in PSC patients, which would result in reduced inhibition or enhanced activation of NK cells circulating in the peripheral blood (61, 62). However, the participation of hepatic NK cells in the progression of PSC is still poorly understood. Our data show significant inhibition of cytotoxicity and IFNG and TNF expression in both hepatic NK cell subpopulations in advanced PSC. Nevertheless, the cNK cells significantly overexpressed the apoptosis mediator TNFSF10 (TRAIL), contradicting recent findings that showed increased TRAIL surface expression in hepatic CD56bright NK cells (rNK cells) in end-stage PSC patients (63). Human cholangiocytes constitutively expressed DR5 (TRAIL receptor), and the importance of TRAIL/DR5 signaling for the progression of cholestatic liver injury has been already shown (64). cNK cells also displayed high levels of the chemoattractants XCL1, XCL2, CCL3, and CCL4. These cells also overexpressed TNFSF9 (CD137L), a receptor involved in T cell activation that has become a significant target in autoimmune disease therapy (65). Therefore, our data suggest that cNK cells are pro-inflammatory in end-stage PSC and may also contribute to cholangiocyte death. Moreover, cNK cells also expressed more exhaustion markers than the NK cells from other groups, markedly upregulation of TIGIT, KLRC1 (NKG2), HAVCR2 (Tim-3), and downregulation of CD226 (DNAM-1), FCGR3A (CD16), and NCR3 (NKp30), which would limit the antitumor and anti-infection potential of NK cells in PSC patients. A remarkable feature of PSC-derived rNK cells was the strong upregulation of the anti-inflammatory PTEN and PPAR signaling. Both signaling pathways affect NK cell cytotoxicity. Increased PTEN signaling is shown to disturb the cytolytic synapse in NK cells (66), while increased PPAR signaling impairs the cytotoxic machinery trafficking to the NK cell-cytolytic synapse (67). Together with the fact that these cells overexpress TGFB1, our results suggest that rNK cells in end-stage PSC contribute to liver fibrosis progression. In addition, the high levels of LGALS3 expressed by the ILC-like cluster may contribute to the progression of autoimmune cholangitis (68).

Here, we characterized the immunological status of intrahepatic NK cells in cirrhotic livers derived from different pathological conditions. In summary, we showed an overall inhibition of hepatic NK cell normal effector functions in the setting of cirrhosis independent of etiology as evidenced by the downregulation of genes encoding important effector molecules such as IFN-γ. However, the overexpression of several chemokines suggests the NK cell involvement in the long-lasting inflammation characteristic of chronic liver disease. Downregulation of several pro-inflammatory pathways in NK cells derived from HCV-infected livers suggests a more profound NK cell functional inhibition in end-stage HCV than in end-stage NASH and PSC. Regarding the subpopulations, rNK may contribute to fibrosis/cirrhosis through a diminished ability to kill activated hepatic stellate cells and increased expression of TGF-β, whereas conventional/infiltrating NK cells display a pro-inflammatory profile. Infiltrating cytotoxic cNKs are likely involved in chronic tissue damage promoting fibrosis progression to cirrhosis, where ADCC may be significant for NASH and TRAIL expression may be important for PSC progression. Thus, our findings support that NK cells are actively involved in the progression of chronic/end-stage liver diseases. While our study highlights etiology-dependent and independent features of rNK and cNK cells, we cannot exclude the contribution of other factors to the observed profiles, such as age, ALT and ALP levels, or previous therapies. Although beyond the scope of this study, future functional and protein analyses would be beneficial to support our scRNA-Seq data. Also, accumulating evidence confirms that dysfunctional NK cells are suitable to be reset and recover their original functions in cancer treatment (69). Therefore, our findings improve the current knowledge of NK cell dysfunction in advanced fibrosis and offer critical information for designing therapeutical strategies targeting these cells for fibrosis prevention and treatment.

## Data availability statement

The data discussed in this publication have been deposited in NCBI’s Gene Expression Omnibus and are accessible through GEO Series accession number GSE217968.

## Ethics statement

The studies involving human participants were reviewed and approved by University of Southern California Institutional Review Board (HS-18-00254). The patients/participants provided their written informed consent to participate in this study.

## Author contributions

LG-M conceived the project. AM-M and LG-M designed the experiments. LD provided clinical evaluation of human samples. AM-M and AH performed the experiments. MS designed the bioinformatic algorithms for data analysis. AM-M and MS analyzed the scRNAseq data. AM-M and LG-M analyzed the data and wrote the manuscript. All authors contributed to the article and approved the submitted version.
