# Supplementary material for: Etiology of end-stage liver cirrhosis impacts hepatic natural killer cell heterogenicity
Source: Front Immunol. 2023 Mar 30;14:1137034. doi: 10.3389/fimmu.2023.1137034 (PMC10098346; doi:10.3389/fimmu.2023.1137034)
Supplement: Supplementary file 1 [file DataSheet_1.docx]

Etiology of end-stage liver cirrhosis impacts hepatic natural killer cell heterogenicity.

Ana C. Maretti-Mira, Matthew P. Salomon, Angela M. Hsu, Lily Dara, Lucy Golden-Mason

Contents List:

Supplementary Figure 1

Supplementary Figure 2

Supplementary Figure 3


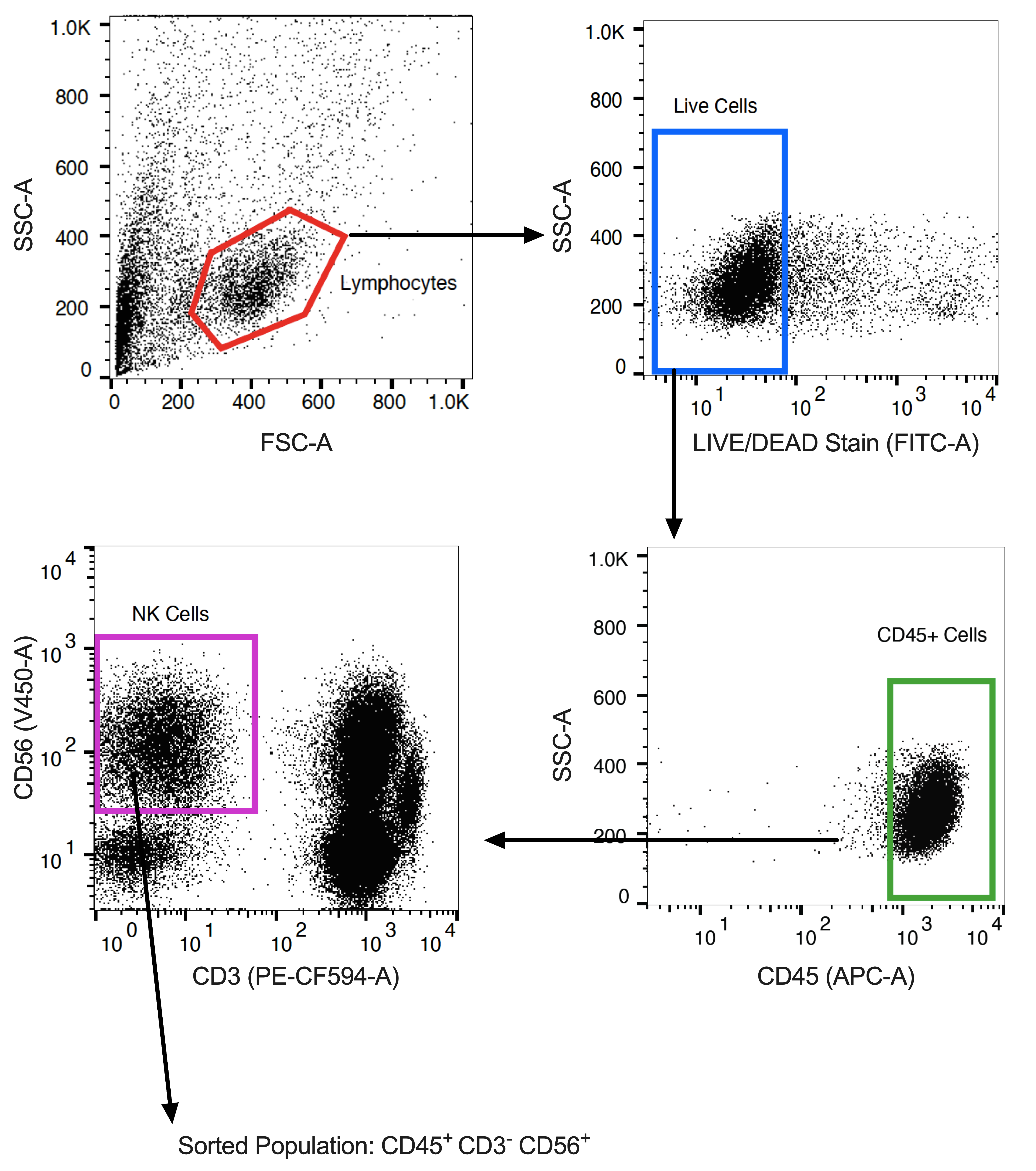


**Supplementary Figure 1:** Total NK cells sorting strategy. Representative graphics from FACS sorting showing the gating strategy used to sort total NK cells from hepatic non-parenchymal single cell suspension. Cells were isolated from human livers with advanced cirrhosis. Cells were sorted in 90% FBS solution at 4˚C.


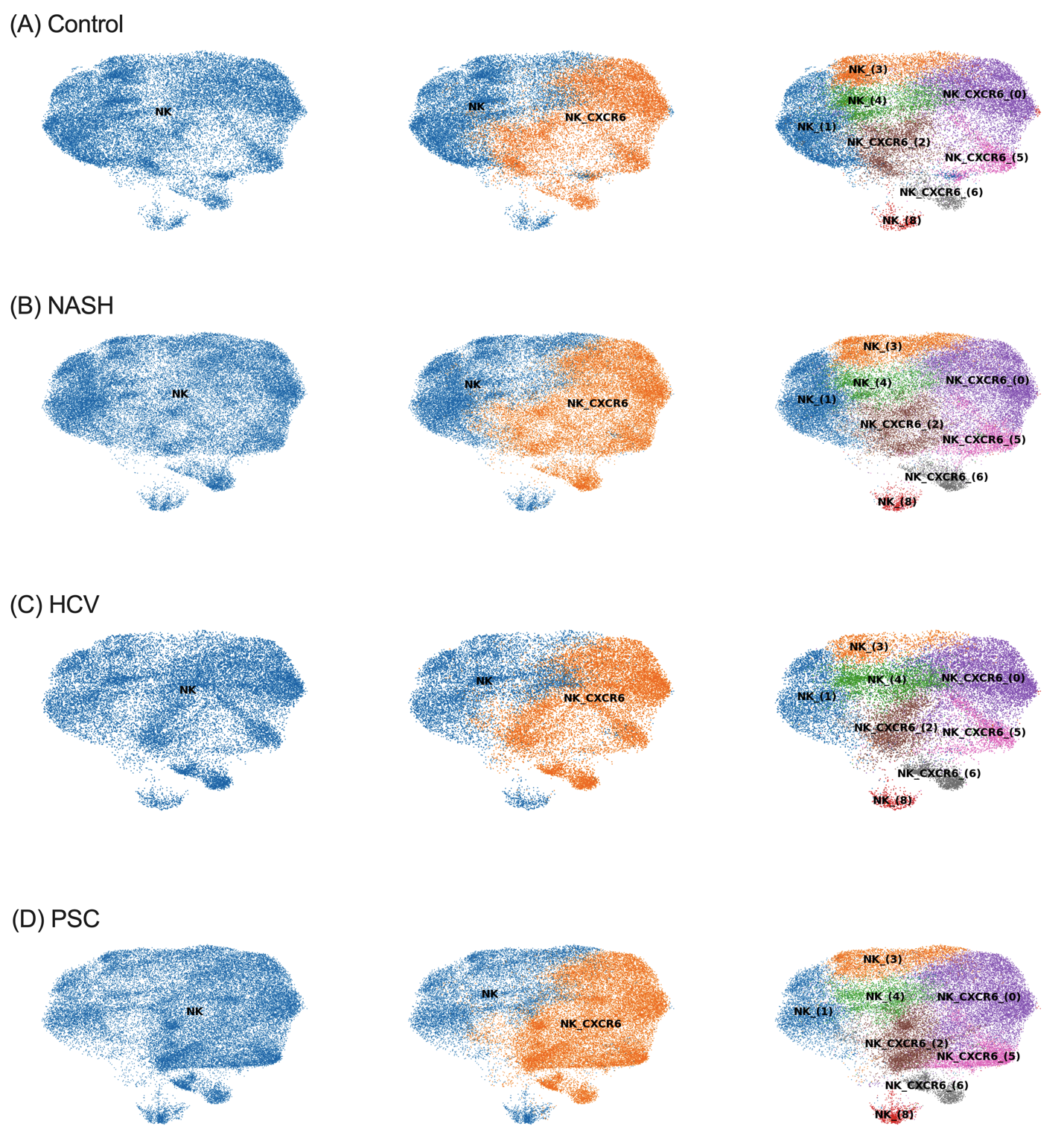


**Supplementary Figure 2:** UMAPs showing the NK cells clustering strategies. The maps showed data integrated by group: (A) non-diseased control, (B) NASH, (C) HCV, and (D) PSC. Legends: NK = cNK cells; NK_CXCR6= rNK cells; NK (1) = C1; NK (3) = C3; NK (4) = C4; NK (8) = C7; NK_CXCR6 (0) = C0; NK_CXCR6 (2) = C2; NK_CXCR6 (5) = C5; NK_CXCR6 (6) = C6.


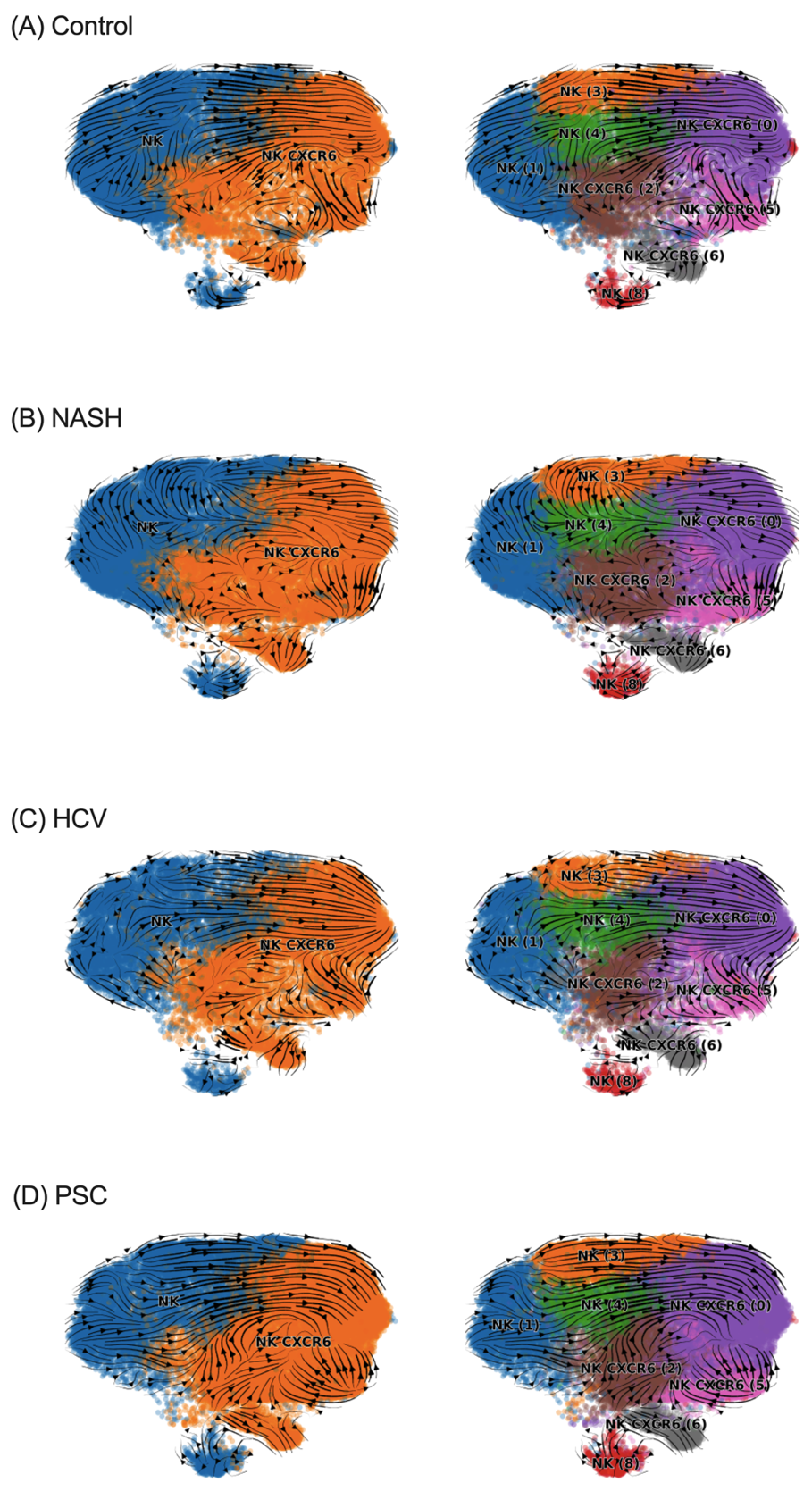


**Supplementary Figure 3:** UMAPs showing the vectors from the RNA velocity analysis performed with the NK cell clusters. The maps showed data integrated by group: (A) non-diseased control, (B) NASH, (C) HCV, and (D) PSC. Legends: NK = cNK cells; NK_CXCR6= rNK cells; NK (1) = C1; NK (3) = C3; NK (4) = C4; NK (8) = C7; NK_CXCR6 (0) = C0; NK_CXCR6 (2) = C2; NK_CXCR6 (5) = C5; NK_CXCR6 (6) = C6.
